# Trends in Severity of Illness on ICU Admission and Mortality among the Elderly

**DOI:** 10.1371/journal.pone.0093234

**Published:** 2014-04-03

**Authors:** Lior Fuchs, Victor Novack, Stuart McLennan, Leo Anthony Celi, Yael Baumfeld, Shinhyuk Park, Michael D. Howell, Daniel S. Talmor

**Affiliations:** 1 Intensive Care Unit, Beth Israel Deaconess Medical Center and Harvard Medical School, Boston, Massachusetts, United States of America; 2 Clinical Research Center, Soroka University Medical Center and Faculty of Health Science, Ben Gurion University of the Negev, Beer-Sheba, Israel; 3 Institute for Biomedical Ethics, University of Basel, Basel, Switzerland; 4 Harvard-MIT Division of Health Science and Technology, Boston, Massachusetts, United States of America; Queen Mary University of London, United Kingdom

## Abstract

**Background:**

There is an increase in admission rate for elderly patients to the ICU. Mortality rates are lower when more liberal ICU admission threshold are compared to more restrictive threshold. We sought to describe the temporal trends in elderly admissions and outcomes in a tertiary hospital before and after the addition of an 8-bed medical ICU.

**Methods:**

We conducted a retrospective analysis of a comprehensive longitudinal ICU database, from a large tertiary medical center, examining trends in patients’ characteristics, severity of illness, intensity of care and mortality rates over the years 2001–2008. The study population consisted of elderly patients and the primary endpoints were 28 day and one year mortality from ICU admission.

**Results:**

Between the years 2001 and 2008, 7,265 elderly patients had 8,916 admissions to ICU. The rate of admission to the ICU increased by 5.6% per year. After an eight bed MICU was added, the severity of disease on ICU admission dropped significantly and crude mortality rates decreased thereafter. Adjusting for severity of disease on presentation, there was a decreased mortality at 28- days but no improvement in one- year survival rates for elderly patient admitted to the ICU over the years of observation. Hospital mortality rates have been unchanged from 2001 through 2008.

**Conclusion:**

In a high capacity ICU bed hospital, there was a temporal decrease in severity of disease on ICU admission, more so after the addition of additional medical ICU beds. While crude mortality rates decreased over the study period, adjusted one-year survival in ICU survivors did not change with the addition of ICU beds. These findings suggest that outcome in critically ill elderly patients may not be influenced by ICU admission. Adding additional ICU beds to deal with the increasing age of the population may therefore not be effective.

## Introduction

The *raison d’être* of intensive care units is to improve clinical outcomes for acutely ill patients. However, reaching a balance between judicious utilization of a limited and high cost resource and providing optimal intensity of care is challenging. This is particularly the case in relation to elderly patients. As the population ages the proportion of very elderly ICU patients is increasing [1]–[5]_._ A recent large retrospective analysis found a yearly increase of 5.6% in very elderly ICU admission rates [4].

While ICU admission can change clinical outcomes for many elderly patients, for others the value of ICU care is questionable [6]. There is positive association between ICU bed availability and ICU admission rates for the very elderly [7]. But the association between hospital ICU capacity and survival benefit for elderly patients is still undefined. A previous European based study found that higher ICU acceptance rate improved survival rates for very elderly patients, especially among those who were previously considered “not sick enough” for ICU admission [7]. This finding highlights the impact of ICU bed capacity and triage on ICU survival. Other studies showed improved outcomes among selected elderly patients once increased intensity of treatment was applied (i.e. renal replacement therapy and vasopressor use) [7]–[13].

The Institute of Medicine’s recent report: *Best Care at Lower Cost* (IOM, 2012), highlights a persistent set of problems within the health care system relating to quality, outcomes, costs, and equity, which, if not addressed, have the potential to negatively affect the performance of the health care system. The report envisions a learning health care system, one that promotes and enables continuous and real-time improvement in both the effectiveness and efficiency of care. Central to this transformation is the utilization of information technology to continuously and reliably capture the care experience, and the use of the data to inform decisions at both the patient and hospital levels.

We hypothesized that an increasing ratio of ICU beds in an institution may lead to a decrease in patient acuity and that by admitting less sick patients to the ICU, a lower crude ICU mortality rate is expected. We sought to study whether mortality and survival in the very elderly were indeed improved by ICU admission before and after the addition of ICU beds at our institution.

## Methods

This is a retrospective observational cohort study, utilizing a massive ICU database collected from electronic medical records. The Multi Parameter Intelligent Monitoring of Intensive Care (MIMIC-II) project [14], [15] was approved by the institutional review boards of the Massachusetts Institute of Technology (MIT) and Beth Israel Deaconess Medical Center (BIDMC) and granted a waiver of informed consent. The MIMIC-II database includes patients admitted between August 2001 and August 2008 and is maintained by researchers at the Harvard-MIT Division of Health Sciences and Technology. It includes physiologic information collected from bedside monitors in adult ICU’s of BIDMC, a large, academic, tertiary medical center in Boston, Massachusetts. The database contains records of demographic and clinical data. Level of acute physiology status on admission was calculated from the data base using SAPS 1 (Simplified Acute Physiology Score) [16] and SOFA (Sequential Organ Failure Assessment) [17]. Further clinical data added to the database included admission and death records, discharge summaries and ICD-9 codes of primary diagnoses of each admission. long-term mortality data derived from the Social Security Administration’s master file of deaths.

### Assembly of the Cohort

Unplanned medical and surgical ICU admissions within the study period of patients older than 65 years were analyzed. The data was extracted from ten bed trauma ICU (TICU), 16 bed surgical ICU (SICU) and two eight bed medical ICUs (MICU). On August 2006 a third eight bed medical ICU was added. By that time, the proportion of ICU to hospital bed was 13%. The SICUs are semi-closed units and the MICUs are closed units. ICU admissions for less than 24 hours, cardiac surgical ICU admissions and non-surgical cardiac ICU admissions were excluded because the focus of this study was on non-planned ICU admission outcomes and differentiating acute admissions from elective admissions in these groups was difficult.

### Statistical Analysis

The cohort was divided into three age groups 65–74 (young elderly), 75–84 (mid elderly) and the age of 85 and above (very elderly). The total of 85 months of the study period was divided equally into three periods of 21 months and one period of 22 months: the first from August 2001 to April 2003, the second from May 2003 to January 2005, the third from February 2005 to October 2006 and the last from November 2006 to August 2008.

The unit of the analysis was admission to ICU. The primary endpoints were 28 days and one year mortality. In order to capture the effect of primary non-planned ICU admission on elderly outcomes (ICU and hospital length of stay and mortality), only the first ICU admission was analyzed. All admissions were analyzed to describe patients’ characteristics. Data were summarized using frequency tables, summary statistics, confidence intervals, and p-values, as appropriate. The preferred method of analyses for continuous variables was parametric. Non parametric analysis methods were used only if parametric assumptions could not be satisfied, even after data transformation attempts. Parametric model assumptions were assessed using Normal-plot or Shapiro-Wilks statistic for verification of normality and Levene’s test for verification of homogeneity of variances. Categorical variables were tested using Pearson’s χ^2^ test for contingency. Kaplan-Meier survival curves with long rank test were built for the analysis of all-cause mortality.

The multivariate analysis for death at 28 days from admission was done using a logistic regression model. The variables were introduced to the model based on the clinical and statistical significance (p value≥0.1 in univariate analysis). The final parsimonious model included the following variables: the age groups 75–84 and over 84 versus the age group of 65–74; DNR status, the SOFA [17] severity score at admission and Elixhauser comorbidity score [18], [19]. The analysis of all patients and landmark one year mortality was done using a Cox proportional hazards survival regression model. For the landmark analysis the model included only patients who survived for 28 days. This type of analysis allows us to assess mortality trends in patients surviving acute period. The variables introduced into the model included the same variables introduced into the logistic regression model.

Time trends were evaluated by fitting a LOcally wEighted Scatterplot Smoothing (LOESS) curve to the monthly data [20]. All p-values reported were rounded to three decimal places. All statistical tests and/or confidence intervals, as appropriate, were performed at α = 0.05 (2-sided). The data was analyzed using SPSS 18 software.

## Results

### Demographics

Between the years 2001 and 2008, 7,265 elderly patients above the age of 65 had 8,916 unplanned admissions to ICU. Patients’ characteristics are presented in Tables 1. Across the four time periods of the study there was an annual increase in elderly ICU admission of 5.6% per year. While the proportion of MICU admissions decreased the proportion of SICU admissions increased (71.8% to 59.9% and 28.2% to 40.1% respectively, p<0.001). There was no change in the median age of the cohort (78 years for all four time periods). The three most prevalent comorbidities remained congestive heart failure, cardiac arrhythmias and hypertension. Although the most prevalent etiology for ICU admission was cardiovascular, the rate dropped from 25.6% of all ICU admissions on first study time period to 19.4% during the final time period (Table 1). Whereas cardiovascular and gastrointestinal reasons for admission remained the first and second most prevalent respectively during the study, infectious (co-second most prevalent during third time period) and trauma prevalence (third most prevalent during last time period) increased.

**Table 1:** Baseline and hospitalization characteristics of ICU admissions, 2001–2008.

Variables			Groups per admission period							
			August 2001–April2003 (n = 1544)		May 2003–January2005 (n = 2070)			February 2005–October2006 (m = 2504)	November 2006–August2008 (n = 2798)	P value
Unit of discharge, n (%)										
Medical			1109 (71.8)		1255 (60.6)			1557 (62.2)	1676 (59.9)	<.001
Surgical/Trauma			435 (28.2)		815 (39.4)			947 (37.8)	1122 (40.1)	
Male gender, n (%)			778 (50.6)		991 (48.1)			1241 (49.6)	1368 (48.9)	.49
Age, years (±SD)			78.09±7.56		78.23±7.73			78.25±7.85	78.91±8.11	.001
Married, n (%)			748 (48.4)		1001 (48.4)			1162 (46.4)	1282 (45.8)	.19
Co morbidities, n (%)										
Elixhauser		5.97±7.20			6.97±7.45		7.53±8.00		6.78±7.94	<.001
Diabetes uncomplicated		257 (16.7)			316 (15.3)		604 (24.2)		687 (24.6)	<.001
Diabetes complicated		67 (4.3)			62 (3.0)		180 (7.2)		156 (5.6)	<.001
Congestive heart failure		455 (29.5)			734 (35.5)		1000 (40.0)		926 (33.1)	<.001
Alcohol abuse		20 (1.3)			44 (2.1)		62 (2.5)		61 (2.2)	.08
Cardiac arrhythmias		447 (29.0)			653 (31.6)		951 (38.0)		1049 (37.5)	<.001
Valvular disease		136 (8.8)			223 (10.8)		342 (13.7)		320 (11.5)	<.001
Hypertension		473 (30.7)			656 (31.8)		1186 (47.4)		1331 (47.6)	<.001
Chronic renal failure		104 (6.7)			199 (9.6)		298 (11.9)		158 (5.7)	<.001
COPD		309 (20.0)			459 (22.2)		637 (25.5)		659 (23.6)	.001
Liver failure		29 (1.9)			71 (3.4)		87 (3.5)		92 (3.3)	.02
Metastatic cancer		84 (5.4)			133 (6.4)		149 (6.0)		196 (7.0)	.18
Psychosis		23 (1.5)			37 (1.8)		74 (3.0)		85 (3.0)	.001
Depression		34 (2.2)			45 (2.2)		104 (4.2)		182 (6.5)	<.001
Drug abuse		2 (0.1)			7 (0.3)		9 (0.4)		1 (0.0)	<.001
Admission source										
Emergency Department	1030 (66.7)	1488 (71.9)			1855 (74.1)		2189 (78.2)			<.001
Other hospital	236 (15.3)	291 (14.1)			293 (11.7)		310 (11.1)			
Other	278 (18.0)	291 (14.1)			356 (14.2)		299 (10.7)			
Proportion of Elderly patients out of all ICU patients (age>65)										
Total number of admissions		3410			4459		5204		6437	<0.001
Proportion of elderly above 65 years of age		45.5%			46.3%		48.4%		43.1%	0.014
Primary admission, n (%)										
Infectious		105 (6.8)			241 (11.7)		376 (15.1)		382 (13.7)	<.001
Cardiovascular		394 (25.6)			465 (22.5)		476 (19.1)		543 (19.4)	
Respiratory		236 (15.3)			280 (13.6)		363 (14.5)		378 (13.5)	
Cancer		144 (9.3)			185 (9.0)		211 (8.5)		250 (8.9)	
Gastrointestinal		247 (16.0)			336 (16.3)		377 (15.1)		421 (15.1)	
Genitourinary		53 (3.4)			65 (3.1)		73 (2.9)		85 (3.0)	
Trauma		194 (12.6)			268 (13.0)		291 (11.7)		412 (14.7)	
Treatment complication		62 (4.0)			108 (5.2)		138 (5.5)		113 (4.0)	
Other		106 (6.9)			118 (5.7)		191 (7.7)		211 (7.5)	
Acuity score on admission										
SOFA		5.82±4.14		5.66±3.85		5.54±3.79			4.87±3.56	<.001
SAPS 1		15.61±5.49		14.86±4.87		14.88±4.82			14.42±4.78	<.001
Intensity of care										
Renal replacement therapy during hospitalization		146 (9.5)		202 (9.8)		267 (10.7)			247 (8.8)	0.16
Mechanical ventilation during hospitalization		845 (54.7)		1185 (57.2)		1406 (56.2)			1254 (44.8)	<.001
Use of vasopressors		513 (33.2%)		605 (29.2%)		689 (27.5%)			627 (22.4%)	<0.001
Don Not Resuscitate (order recorded during ICU admission)		496 (34.1)		720 (35.8)		898 (36.6)			887 (34.3)	0.25
MV duration (hours)		109.17 (32–313.88)		107.50 (36.0–280.13)		88.63 (32.25–266.13)			59.75 (21.55–175.08)	<0.001
ICU LOS (days)		5.30 (2.44–12.05)		5.68 (2.99–11.97)		4.97 (2.74–9.91)			4.42 (2.44–8.64)	<0.001

All elderly admissions (n = 8916).

ICU, intensive care unit; COPD, chronic obstructive pulmonary disease,SOFA, sequential organ failure assessment; SAPS, simplified acute physiology score.

### Acuity of ICU Admissions

**Figure 1:**
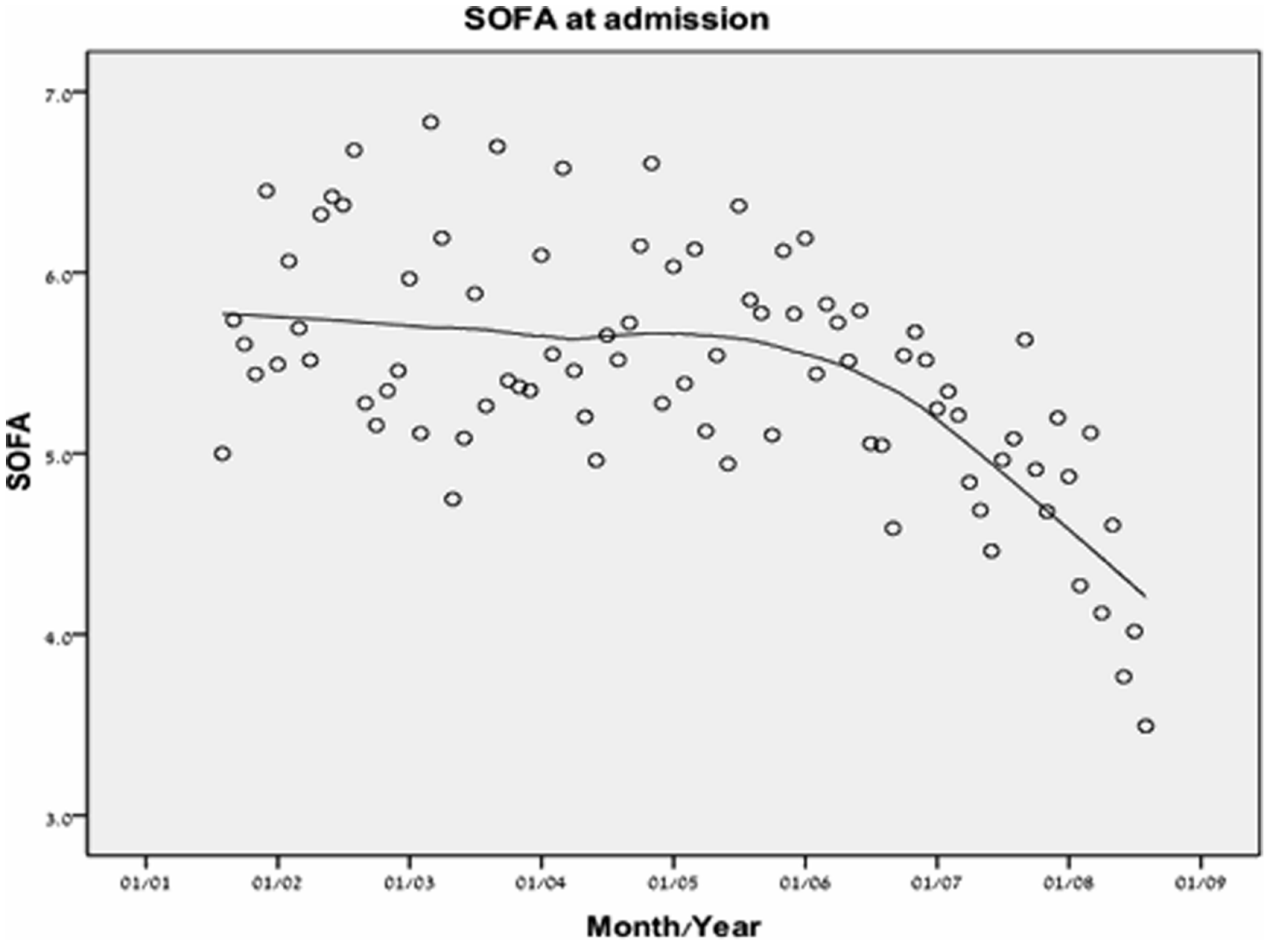
Severity of disease on admission presented by LOcally wEighted Scatterplot Smoothing (LOESS) curves. N = 8,916.

The severity of disease on ICU admissions, as reflected by the first SOFA score, decreased in a non-linear manner with significant decline between the third and the fourth time periods (Table 1, Figure 1). By subgroup analysis, this trend was true in both MICU and SICU patients.

### Admission Trends

**Figure 2:**
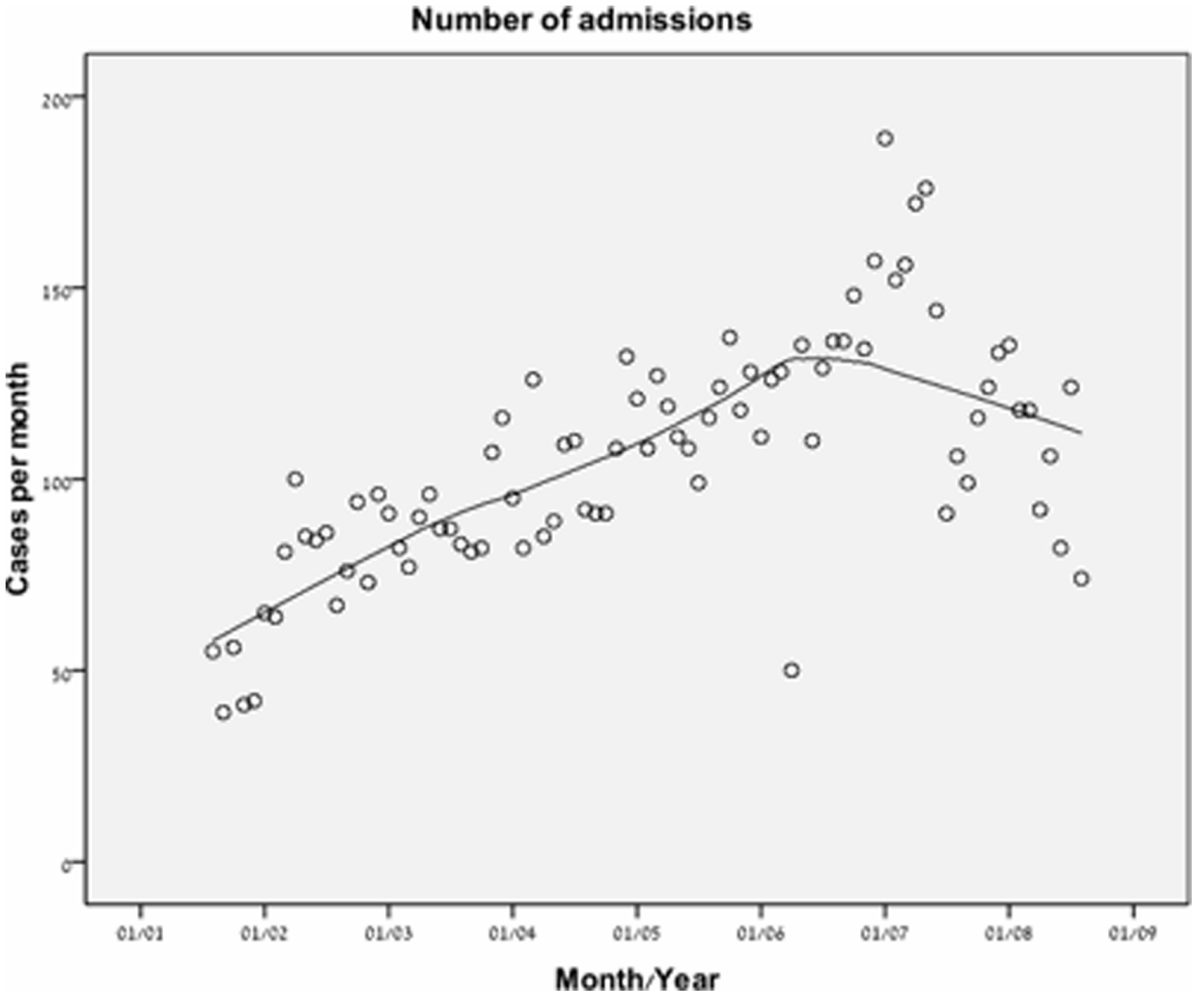
Trend for number of ICU admissions of patients older than 65 years presented by LOcally wEighted Scatterplot Smoothing (LOESS) curves. N = 8,916.

**Figure 3:**
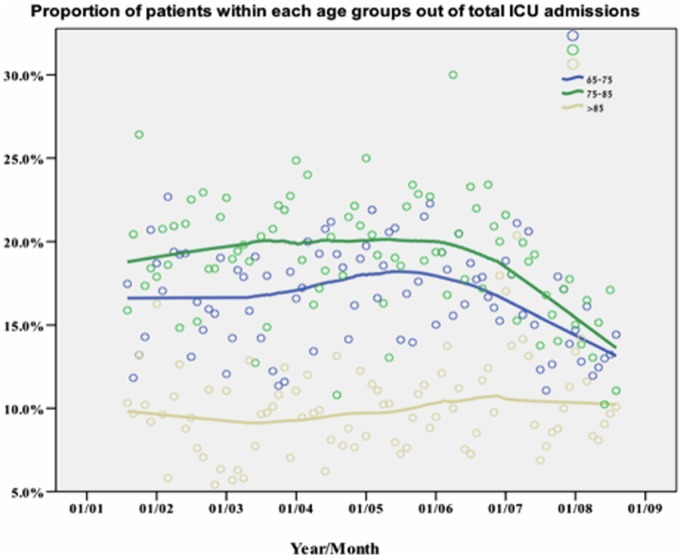
Proportion of patients within each age subgroup out of total ICU admissions presented by LOcally wEighted Scatterplot Smoothing (LOESS) curves. N = 8,916.

The crude number of admissions to the ICU increased in a nonlinear fashion (Figure 2). There was a steady increase in elderly ICU admission rates in the first three time periods and plateau thereafter (from 73.5 cases per month in the first time period to 127.2 cases per month in the fourth time period). Although elderly admissions increased at an annual rate of 5.6% per year, the proportion of the very elderly (age over 85) patients out of all ICU patients remained similar throughout the study (about 10% a year) (Figure 3). The final time period showed a decrease in the proportion of elderly patients (43.1% of all ICU patients from 45.5% of all ICU patients) (Figure 3 and Table 1).

### Mortality Trends

**Figure 4:**
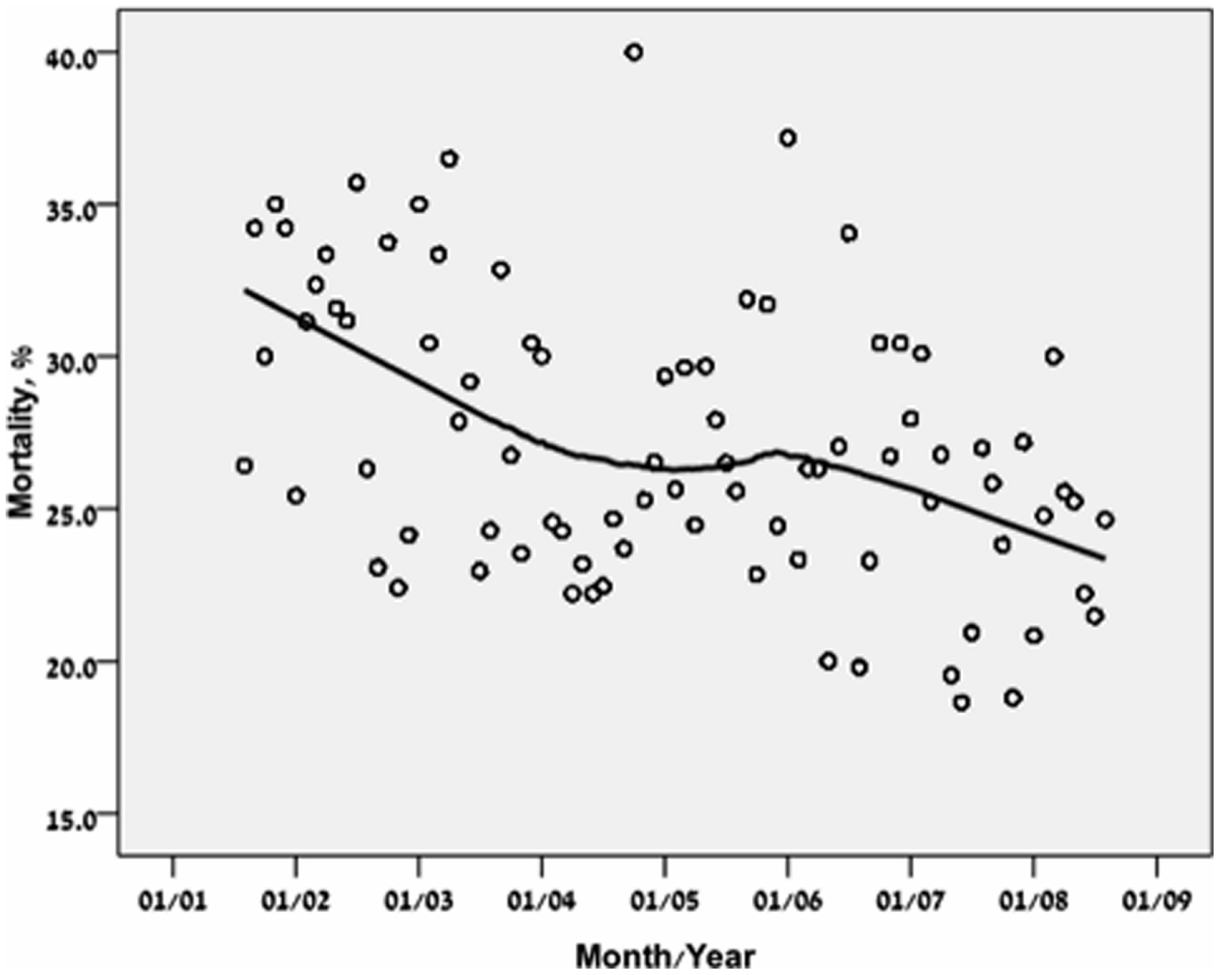
28 days mortality of first ICU admission (7,265 subjects) presented by LOcally wEighted Scatterplot Smoothing (LOESS) curves.

**Figure 5:**
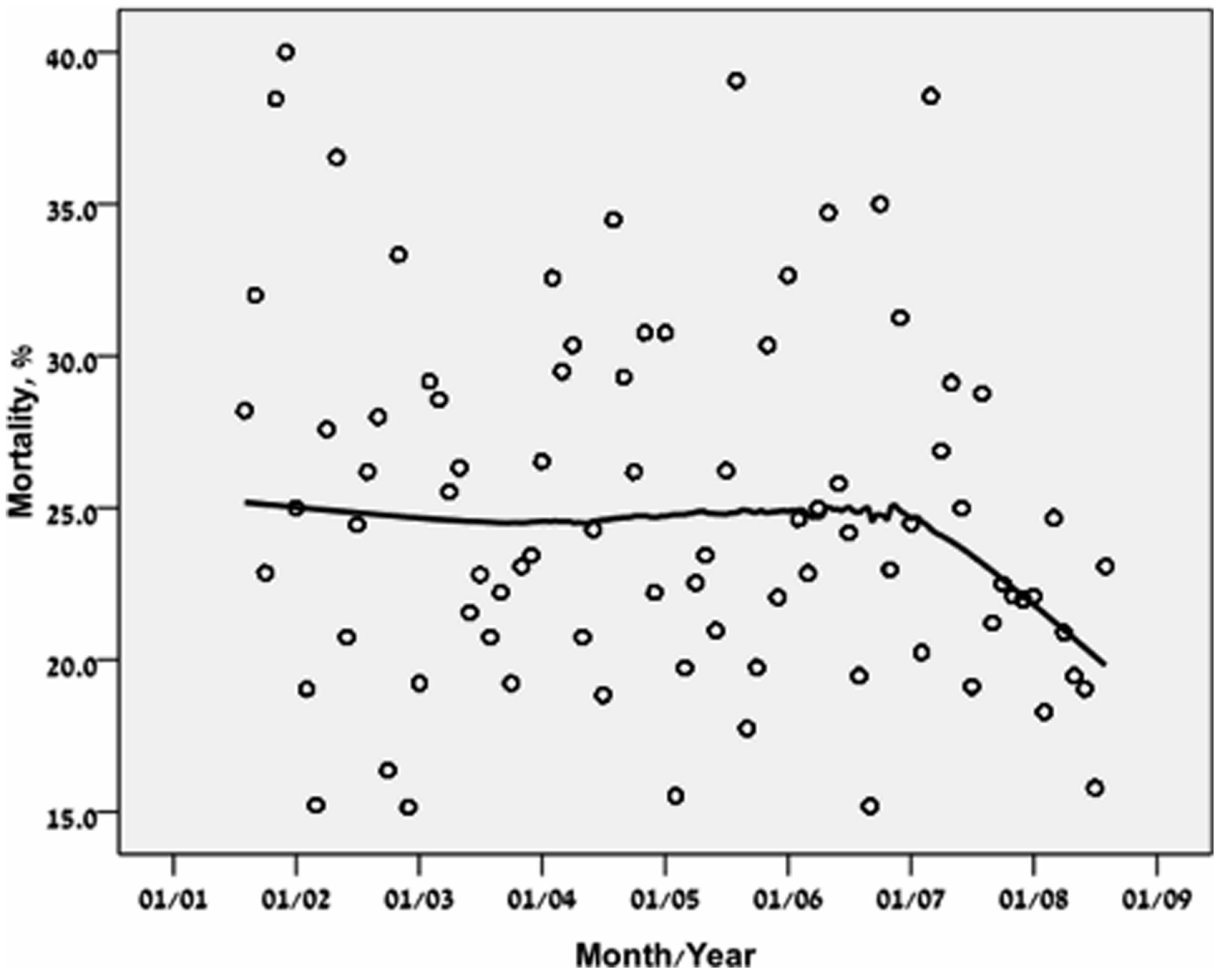
One-year mortality in 28 days survivors of first admission (5,317 subjects) presented by LOcally wEighted Scatterplot Smoothing (LOESS) curves.

**Figure 6:**
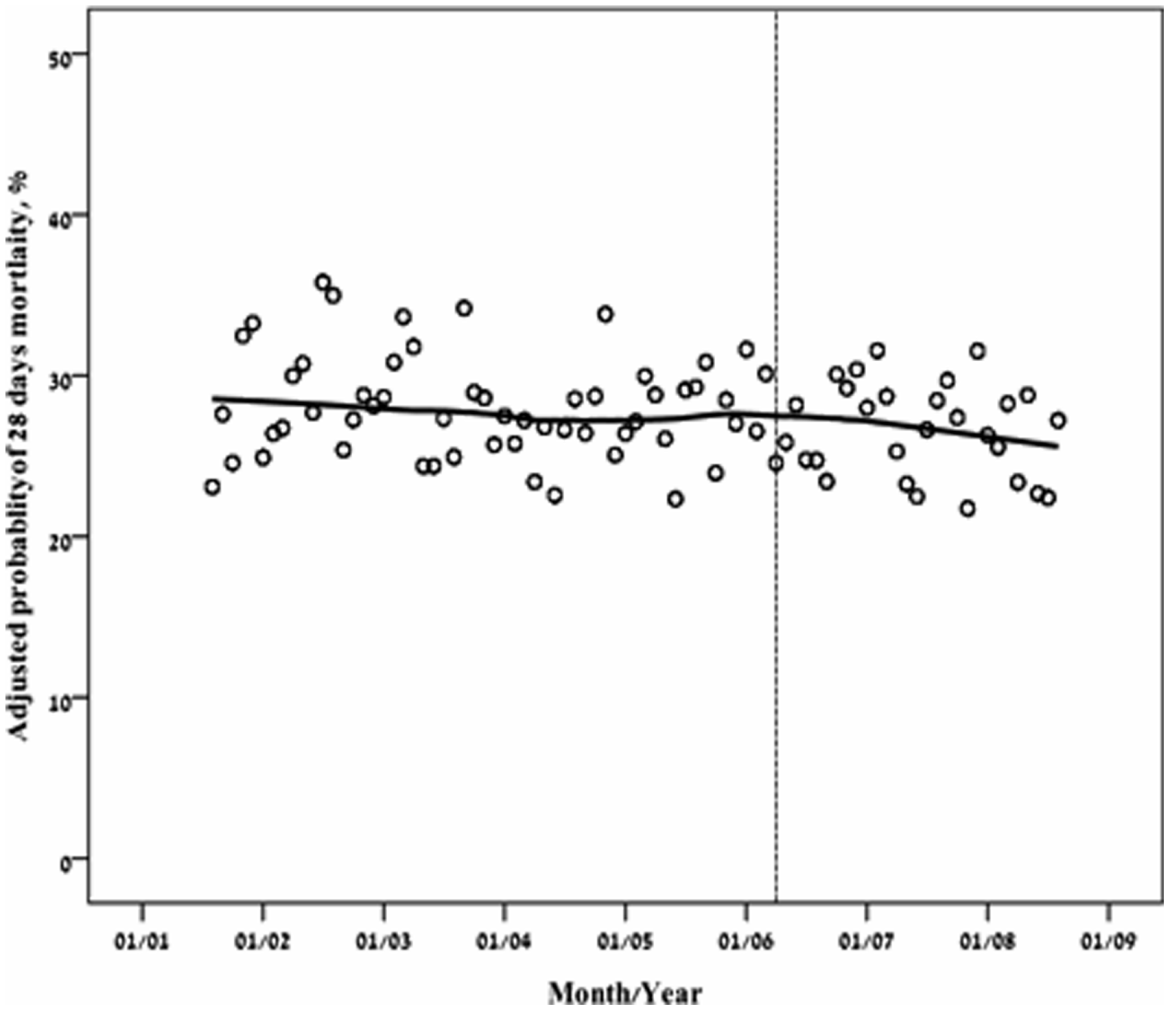
Adjusted 28 days mortality of first ICU admission (7,265 subjects) presented by LOcally wEighted Scatterplot Smoothing (LOESS) curves.

For these analyses, only the first ICU admission (N = 7265) was used for each patient. Age was directly associated with ICU, in hospital, 28 days and one year mortality rate (Table 2). The crude short (28 days) and long term (one year) post ICU mortality rates continuously decreased through the years (Figure 4 and 5). Logistic regression analysis (Table 3, Figure 6) showed however that adjusted for baseline characteristics (SOFA, Elixhauser score, DNR code status and age) there was an improvement in 28 day mortality during the study period (OR 0.93,p = 0.0.01). Cox regression analysis and land mark analysis for one year mortality in 28 days survivors (Tables 4) failed to show improved survival through the study period (HR 0.96, p = 0.06). As expected, age (OR 1.23 per year, P<0.001), SOFA score (OR 1.21 per point, P<0.001), DNR code status (OR 6.07, P<0.001), and Elixhauser comorbidities score (OR 1.06 per point, <0.001) were found to be independent risk factors for 28 days mortality. All remained significant for one year mortality (Tables 4). During the study period from 2001 through 2008, hospital mortality rate at BIDMC remained the same (Table 5, P = 0.43).

**Table 2:** Clinical outcomes (n = 7265).

		Age groups			P value
		65–74 n = 2585 (35.4%)	75–84 n = 3003 (41.1%)	Over 84 n = 1677 (23.5%)	
LOS in days (median, IQ)					
	Hospital	9 (5–15)	8 (5–14)	7 (4–11)	<.001
	ICU	2.41 (1.22–5.04)	2.24 (1.27–4.58)	2.07 (1.13–3.76)	<.001
Mortality, n (%)					
	In-Hospital	486 (18.8)	706 (23.5)	468 (27.9)	<.001
	In-ICU	285 (11.0)	423 (14.1)	245 (14.6)	<.001
	Mortality 28 days	528 (20.4)	840 (28.0)	580 (34.6)	<.001
	1 year mortality	937 (36.2)	1377 (45.9)	941 (56.1)	<.001

LOS length of stay; ICU intensive care unit.

**Table 3:** Logistic Regression Models of 28 days Survival of ICU Patients.

Variables	Odd Ratio	95% CI	P value
Time Period	0.93	0.88–0.98	0.01
Age, per group	1.23	1.14–1.32	<0.001
SOFA, per point	1.21	1.19–1.22	<0.001
DNR	6.07	5.40–6.82	<0.001
Elixhauser score, per point	1.06	1.05–1.08	<0.001
Surgical/Trauma ICU admission (versus Medical ICU)	1.19	1.06–1.35	0.01
COPD	0.90	0.79–1.03	0.13
CHF	0.86	0.76–0.98	0.02
DM	1.04	0.91–1.20	0.57
CRF	0.86	0.71–1.03	0.10

First admissions (n = 7265).

SOFA, sequential organ failure assessment; DNR, do not resuscitate; COPD, chronic obstructive pulmonary disease; CHF, congestive heart failure; DM, Diabetes Melitus; CRF, chronic renal failure.

**Table 4:** Cox Regression Models for 1-Year Survival of ICU Patients.

Variables	Hazard Ratio	95% CI	P value
Time period	0.96	0.91–1.003	0.06
Age, per group	1.22	1.14–1.31	<0.001
SOFA, per point	1.04	1.02–1.06	<0.001
DNR	1.84	1.65–2.06	<0.001
Elixhauser score, per point	1.06	1.06–1.07	<0.001
Surgical/Trauma ICU admission(versus Medical ICU)	0.88	0.79–0.98	0.02
DM	1.11	0.98–1.25	0.10
CHF	0.89	0.80–0.99	0.03
RF	1.001	0.85–1.18	0.99
COPD	1.07	0.96–1.19	0.27

Landmark analysis of 28 day survivors, n = 5317.

SOFA, sequential organ failure assessment; DNR, do not resuscitate; ICU, intensive care unit; COPD, chronic obstructive pulmonary disease; CHF, congestive heart failure; DM, Diabetes Melitus; CRF, chronic renal failure.

**Table 5:** Hospital mortality rate per study years (adults).

Year	Average age	Discharges from hospital	Died during hospital admission	Mortality rates	P value
2001	55.6	31669	743	2.35%	0.43*
2002	55.1	32152	653	2.03%	
2003	54.8	33168	654	1.97%	
2004	55.0	33700	634	1.88%	
2005	55.5	33091	747	2.26%	
2006	55.4	34216	705	2.06%	
2007	56.0	35992	751	2.09%	
2008	56.2	37082	692	1.87%	

All comers.

*Between all mortality rates.

Among the elderly, SICU admission compared with MICU admission was found to be a significant short term mortality risk factor (OR 1.19 p = 0.01; Table 3) but not long term mortality risk factor once you survived ICU admission (OR 0.88, p = 0.02; Table 4).

## Discussion

Our findings demonstrate a temporal decrease in severity of disease on ICU admission between the years 2001 and 2008, more so after the addition of additional medical ICU beds. While crude mortality rates and adjusted 28- day mortality rates decreased over the study period, one-year survival in ICU survivors did not change with the addition of ICU beds. We found that below a certain level of acuity of illness, expanding the ICU bed capacity led to decreased acuity on ICU admission and decreased utilization of mechanical ventilation, vasopressors and renal replacement therapy but did not reduce adjusted mortality rates among those who were admitted to the ICU. Hospital mortality during the study period remained steady. While the mean age of patients admitted to the ICU increased, the mean age of the patients admitted to the hospital was unchanged (Table 5). This suggests that elderly patients were disproportionately admitted to the ICU. The addition of an 8 ICU beds, did not impact either the adjusted hospital or ICU one-year mortality. These findings suggest that outcome in critically ill elderly patients may not be influenced by ICU admission and that adding additional ICU beds to deal with the increasing age of the population may therefore not be effective.

### Severity of Illness on ICU Admission and Mortality Rates

After the addition of eight ICU beds in 2006, the severity of disease scores (by SOFA and SAPS scores) trended abruptly downwards and mortality trend followed (Figures 1 and Table 1). This picture may be misleading; one can assume that by reducing the admission threshold, mortality can be reduced in a similar manner. However, after adjustment for severity of disease a short term mortality reduction but not long term mortality reduction was observed (Tables 3 and 4, Figure 6).

### Intensity of Care and Elderly ICU Survival

Previous studies have shown that admitting sicker elderly patients to the ICU and increasing the intensity of care may improve survival. Lerolle et al. showed a 300% mortality rate reduction by admitting sicker elderly patients (80 years and above) and by providing more aggressive treatment (higher utilization rate of vasopressor, mechanical ventilation and renal replacement therapy) [8]. Other studies have shown the same trend [9], [11].

In our study, the intensity of care was characterized by abrupt reduction in mechanical ventilation rate and time on the ventilator (52.6% to 44.8%, p<0.001 and 109 hours to 59 hours respectively ) and vasopressors utilization (27.5% to 22.4%, p<0.001) after the addition of an eight bed MICU. This reduced intensity of care was not associated with lower mortality rates. The difference in our findings is likely a reflection of the lower severity of illness for our ICU patients compared to those studied by Lerolle et al. (mean SAPS score of 14.9 versus 50 respectively). They have found an increase in intensity of care and improved outcomes once elderly admission threshold was liberalized [8].

Reaching a balance between judicious resources utilization and providing optimal intensity of care is challenging. Survival benefit as well as costs needs to be factored when the proportion of ICU to hospital beds is being addressed, especially in light of recent data that shows that the number of ICU beds is increasing over time [21]. High rejection rate of elderly patients from the ICU may result in worse outcomes. The European based Eldicus trial showed that ICU refusal rate increased with patient’s age and by liberalizing elderly ICU admission threshold to a certain degree improved survival [7]. Other studies showed that higher intensity of care also improved survival [8], [9], [11].

This study describes the other side of the spectrum. In our medical center, as opposed to those that participated in the Eldicus trial, there is higher ratio of ICU to hospital beds and no ICU refusal policy resulting in a higher proportion of elderly patients that are admitted to the ICU (10% vs. 3.3% of total ICU patients over age 85). This higher admission rate also translated to a lower severity of disease score on ICU admission (SAPS score of 14.9 compared to 33.0). Between 2001 and 2008, after adjustment for severity of disease, there was a temporal improvement only in short term mortality rate, but we could not find a change in adjusted elderly ICU survivors mortality rates. Adjusted hospital mortality, likewise did not improve. This raises the question whether consistently increasing the number of ICU beds and further lowering ICU admission threshold, improves patient outcomes, or whether there exists a saturation point above which increasing ICU bed capacity maybe wasteful.

It is unethical to conduct a study where patients are randomized with regard to ICU versus regular ward admission. But a cohort study that compares outcomes for patients who were admitted to the ICU (in a hospital with low threshold for ICU admission) versus a comparable cohort of patients that are admitted to a ward (in a hospital with higher threshold for ICU admission) may help to better define the patient population that will benefit the most from an ICU admission. A recent paper by Wunsch, discusses the question: “Is there a Starling curve for intensive care?” [22]. Our findings are consistent with her conclusion: Likely there is, but the subgroup of patients that will benefit from an ICU admission is yet to be defined. Future studies should focus on describing this threshold point.

The strengths of this study include the combination of large and comprehensive ICU data base and the length of follow up. There is scarce literature on longitudinal ICU admission trends. To our knowledge, this is one of the largest studies in the last decade examining trends in the characteristics and outcomes of the elderly and very elderly ICU population. Also, due to the liberal ICU admission policy and uncommon high ICU to hospital bed ratio, we were able to study a spectrum of less sick ICU patients. The study period captured the addition of an 8-bed medical ICU, allowing us to compare adjusted hospital and ICU mortality rate before and after the change.

Weaknesses of this study relate to this being a single center study. Also, we had no data on non ICU patients, so there was no matched control group of very elderly patients who were not admitted to the ICU. It is also acknowledged that quality of life assessment post ICU admission is an important end point, which this study did not address.

Finally, we do not know of any other changes during the study years that can explain the abrupt reduction in severity of disease on ICU admission other than the addition of the 8 MICU beds, but we are aware that there may be non-captured covariates. In order to adjust for these un-captured covariates we have included the 4 predefined time periods as independent variables in our model.

## Conclusions

In our high capacity ICU bed hospital, decreased severity of disease on ICU admission was not associated with lower elderly ICU survivors adjusted mortality rates. Increasing the number of ICU beds was associated with a reduction of the acuity of illness of patients at the time of ICU admission as well as the intensity of care provided to elderly ICU patient population and no change in overall hospital mortality.”

Further research is needed to better define the elderly patient population that will most benefit from ICU admission.

## Acknowledgments

**Location of study:** The study was performed at Beth Israel Medical Center, Boston, MA, USA.
